# Supplementary material for: Patterns of Intron Gain and Loss in Fungi
Source: PLoS Biol. 2004 Nov 30;2(12):e422. doi: 10.1371/journal.pbio.0020422 (PMC532390; doi:10.1371/journal.pbio.0020422)
Supplement: Table S1 — Also available at http://genes.mit.edu/NielsenEtAl/. (4.3 MB ZIP). [file pbio.0020422.st001.zip › NielsenEtAl/html/1360.html]

AN1810.1.NCU00194.1.MG06392.1.FG05546.1


```
 CLUSTAL W (1.82) Multiple Sequence Alignments - Introns Inserted


Sequence 1: NCU00194.1	452 aa
Sequence 2: MG06392.1	409 aa
Sequence 3: FG05546.1	437 aa
Sequence 4: AN1810.1	454 aa
Alignment Length: 468 aa
Number Identitical Residues: 237 aa
Alignment Score (without introns) 11950


MG06392.1 	---MTPSATNG-------DAAAASSRFHASSTKSAIAAENEYAAHNYHPIPVVFARAQGV
NCU00194.1	MAPLADTASNGGSTLSLDHLITGSDKYHAAATNVAIAAENEYAAHNYHPLPVVFAKAQGV
FG05546.1 	---MSP------------HAEKTVSRFHATSTDKAIAIEEEYAAHNYHPLPVVFSRAEGV
AN1810.1  	---MTSNGTNG-----------SATAYHASSTQEAIQAENDFAAHNYHPLPVVFARAQGT
          	   :: ..:..               :**::*. **  *:::*******:****::*:*.

MG06392.1 	NVWDPEGKHYLDLLSAYSAVNQGHCHPELIKALAEQAGRLTLSSRAFYNDVFPVWAAKVR
NCU00194.1	HVWDPEGKHYLDFLSAYSAVNQGHCHPELVKALTEQASRLTLSSRAFHNDVFPVWAAKVR
FG05546.1 	NVWDPEGNHYYDFLSAYSAVNQGHCHPELIKTLTEQAGRLTLSSRAFYNDVFPKWAEKVR
AN1810.1  	SVWDPEGRHYLDFLSAYSAVNQGHCHPKLVAALVDQASRLTLSSRAFYNDVFPKFAEMVT
          	 ******.** *:**************:*: :*.:**.*********:***** :*  * 

MG06392.1 	DLFGYDMVLPMNTGAEAVETAIKIARKWAYKVKGVPQGKAHVFSVADNFHGR-~------
NCU00194.1	SVFGYDMVLPMNTGAEAVETAIKIARKWAYKVKGVPHDKALVFSAADNFHGRT0MTAISL
FG05546.1 	SVFGYDMVLPMCTGAEAVETAIKIARKWAYKVKGVPQEKAWIFGVSENFHGRT0MTAITL
AN1810.1  	KYFGFDMVLPMNTGAEAVETGIKIARKWGYKVKGIPENEAIILSAENNFHGRT0MAAISL
          	. **:****** ********.*******.*****:*. :* ::.. :*****:  :: : 

MG06392.1 	--------------------------T0IRYNNISDLEIVLEAHGAETAAFIVEPIQGEA
NCU00194.1	SVDPESRDNYGPYVPQIGAVSPSTGKT~IRYNNIADLEEVLEAHGKETAAFIVEPIQGEA
FG05546.1 	SVDPESRENYGPYVPNVGAINPTTGKA~IRYNNIADLEEVLEAHGKETAAFICEPIQGEA
AN1810.1  	SSDPESRENYGPYVPNIGCTIPGTEKP~ITYNDKAALREAFEKAGSNLAAFLVEPIQGEA
          	: ...: .. ..  .. ..  . : .. * **: : *. .:*  * : ***: *******

MG06392.1 	GVVVPDDDYLAKVHALCKKHNVLFICDEIQTGIARTGKMLCCNWAGIKPDIVTLGKAISG
NCU00194.1	GVVVPDEDYLQKVSALCKKHNVLFICDEIQTGIARTGRMLCSDWANIKPDIITLGKAISG
FG05546.1 	GVVVPDDDYLAKVQALCTKHNVLFICDEIQTGIGRTGRMLCSEWANIKPDLVTLGKAISG
AN1810.1  	GIIVPDDDYLQLARSLCDQHNVLLICDEIQTGIARTGKLLCHEWSGIKPDMVLLGKAISG
          	*::***:***  . :** :****:*********.***::** :*:.****:: *******

MG06392.1 	GMYPVSCVLADKDVMMVVEPGTHGSTYGGNPLGCAVSIRALELVEEGKLADQADHLGRIF
NCU00194.1	GMYPVSAVLADKEIMLVVEPGTHGSTYGGNPLGCAVSLRALELVEEEGLVEKAEKLGQIF
FG05546.1 	GMYPVSCVLSSKEVMLVVEPGTHGSTYGGNPLGSAVSIRALEIMEEEDLTAKADKLGNMF
AN1810.1  	GMYPVSCVLGRKDVMLTVEPGTHGSTYGGNPLACAVAIRALEVVQEENMVERAEKLGQAF
          	******.**. *::*:.***************..**::****:::*  :. :*::**. *

MG06392.1 	REGVEAFKSPIVQQVRGKGLLNAVVIDESAAGGRTAWDLCMLLKSKGLL0AKPTHGNIIR
NCU00194.1	REGVEAFKSPIVKLVRGKGLLNAVVIDESAAAGRTAWDLCILLKEKGVL0AKPTHGNIIR
FG05546.1 	RDGLKALNTPILKVIRGKGLLNAVVIDESAANGRTAWDLCLLLKSKGLL~AKPTHGDIIR
AN1810.1  	RSGLEAIQNPIIQTVRGKGLLNAIVIDESKTNGHTAWDLCMLMKEKGLL0AKPTHQNIIR
          	*.*::*::.**:: :********:***** : *:******:*:*.**:* ***** :***

MG06392.1 	FAPPLIITEEELKKALSIIGEALTELPTAEKGDGH----------------
NCU00194.1	FAPPLVITEEELRGAIKTFGEALQELPTVTKSEGH----------------
FG05546.1 	FAPPLVISEEDLKKGLNIIAEAIKELPTVEPAKHH----------------
AN1810.1  	LAPPLVITEEEIAKALEIIKAAVAELPNLKGAAEDKVVPPPEKKVKITLEN
          	:****:*:**::  .:. :  *: ***.   .  ..  ...... . : ..
```
